# Analysis of Microcystins in Cyanobacterial Blooms from Freshwater Bodies in England

**DOI:** 10.3390/toxins10010039

**Published:** 2018-01-11

**Authors:** Andrew D. Turner, Monika Dhanji-Rapkova, Alison O’Neill, Lewis Coates, Adam Lewis, Katy Lewis

**Affiliations:** 1 Centre for Environment, Fisheries and Aquaculture Science, Barrack Road, The Nothe, Weymouth, Dorset DT4 8UB, UK; monika.dhanji.rapkova@cefas.co.uk (M.D.-R.); Alison.oneil@cefas.co.uk (A.O.); Lewiscoates@cefas.co.uk (L.C.); Adam.lewis@cefas.co.uk (A.L.); 2 Environment Agency, Horizon House, Deanery Rd, Bristol BS1 5AH, UK; katy.lewis@environment-agency.gov.uk

**Keywords:** cyanobacteria, microcystins, freshwater blooms, LC-MS/MS, toxin profiles, First systematic screen of microcystins in English water bodies, showing variable toxin profiles and some environmental dependencies. Results show importance of routine toxin testing to supplement microscopic identification of cyanobacteria.

## Abstract

Cyanobacterial blooms in freshwater bodies in England are currently monitored reactively, with samples containing more than 20,000 cells/mL of potentially toxin-producing species by light microscopy resulting in action by the water body owner. Whilst significantly reducing the risk of microcystin exposure, there is little data describing the levels of these toxins present in cyanobacterial blooms. This study focused on the quantitative LC-MS/MS analysis of microcystins in freshwater samples, collected across England during 2016 and found to contain potentially toxin-producing cyanobacteria. More than 50% of samples contained quantifiable concentrations of microcystins, with approximately 13% exceeding the WHO medium health threshold of 20 μg/L. Toxic samples were confirmed over a nine-month period, with a clear increase in toxins during late summer, but with no apparent geographical patterns. No statistical relationships were found between total toxin concentrations and environmental parameters. Complex toxin profiles were determined and profile clusters were unrelated to cyanobacterial species, although a dominance of MC-RR was determined in water samples from sites associated with lower rainfall. 100% of samples with toxins above the 20 μg/L limit contained cell densities above 20,000 cells/mL or cyanobacterial scum, showing the current regime is suitable for public health. Conversely, with only 18% of cell density threshold samples having total microcystins above 20 μg/L, there is the potential for reactive water closures to unnecessarily impact upon the socio-economics of the local population. In the future, routine analysis of bloom samples by LC-MS/MS would provide a beneficial confirmatory approach to the current microscopic assessment, aiding both public health and the needs of water users and industry.

## 1. Introduction

Cyanobacteria are a well-known group of photosynthetic bacteria which can be found globally, being distributed from polar regions to the tropics [1,2]. Within this broad classification of organisms there exists a range of species which can cause problems for humans, via mechanical clogging of filter equipment [3], creation of hypoxic water bodies leading to death of aquatic organisms [2], reduction in recreation and tourism [4] and via the production of toxins capable of affecting humans directly [4,5,6,7]. Factors influencing cyanobacteria growth include light intensity, water temperature and nutrient (phosphate and nitrate) availability [8,9,10,11], with the latter typically resulting from increased levels of precipitation linked to agricultural and industrial nutrient run-off promoting eutrophication [12]. Of those genera of cyanobacteria containing species known to produce toxins, *Microcystis* occurs commonly around the globe with issues caused by toxic *Microcystis* having been reported from Australia, Brazil, China, Portugal, Sweden and the USA [4,5,11,12,13] amongst others. In the UK, in addition to *Microcystis* spp., other cyanobacteria genera were reported, including *Oscillatoria*, *Plantothrix*, *Anabaeana*, *Pseudoanabaena*, *Aphanizomenon*, *Snowella* and *Gomphospaeria* [8,14,15]. 

Microcystins (MCs) are the primary toxins associated with cyanobacteria, including *Microcystis* spp., and they represent one of the most common and most studied groups of cyanotoxins [1,4]. MCs are cyclic heptapeptides with more than 240 known analytes which vary in toxicity [4]. In humans and other mammals these toxins act as hepatotoxins, inhibiting protein phosphatases [11] and are known to be tumorigenic [16], neurotoxic [17] and genotoxic [18]. Routes of exposure for humans include intravenous injection [5], skin contact [19], inhalation [19] and ingestion, either directly in the form of drinking or unintentionally through recreational water activities [20,21], or potentially via a food vector [19,22]. More detailed information on human intoxications is presented in the recent review by [4], based on a wealth of global cases. Testing methods have been developed to allow for the detection of these toxins, primarily in water. In turn this has led to the World Health Organisation (WHO) recommendation of a drinking water guideline value for microcystin-LR (MC-LR), of 1 μg/L for life long consumption [23]. To date this remains the only cyanotoxin group which has received such guidance in part due to being well studied. Consequently, the MCs have become the toxin group which has received the most attention as a framework for assessing risk.

Within the United Kingdom (UK) there are few reported cases of microcystin related intoxications originating from cyanobacteria. These cases primarily relate to intoxications of animals, such as sheep and dogs [13,24], after drinking *Microcystis* contaminated lake water. However, cases of human intoxications have been reported following exposure to *Microcystis* and *Plantothrix* blooms [6,20]. 

Although, to date, cyanobacterial blooms in the UK have not resulted in widespread intoxications of humans through direct exposure or via drinking water, the presence of these harmful species within UK water bodies does not rule out the possibility of future contaminations. Work conducted in the late 1980’s showed a prevalence of microcystins in 68% of 91 bloom sites surveyed within the UK [13], suggesting that the potential for microcystin intoxications exists across the country. The current stance by Water UK is that microcystins pose a limited threat to UK drinking water supplies [25,26]. Direct contact through recreational use of contaminated water bodies remains a potential route for exposure. In recent years, open-water swimming has become a highly popular sport, with thousands of swimmers involved in more than 170 mass events each year in the water bodies around the country [27,28]. Over the past twenty years, various agencies have carried out assessments of freshwater bodies in the UK to determine the presence of cyanobacterial blooms [14,29,30,31,32]. Blooms were identified in a significant number with many of these linked to eutrophication. As such, in England the Environment Agency (EA), and in Scotland the Scottish Environmental Protection Agency (SEPA) both operate programmes whereby water samples are taken from locations in response to the occurrence of visual blooms of freshwater algae. Neither organisation currently operates a routine monitoring programme to establish the frequency and intensity of cyanobacterial blooms [14,33]. Water samples are taken and processed using light microscopy to determine the presence and density of potentially harmful cyanobacterial species. In England as well as Scotland, water samples containing cyanobacterial species are enumerated to quantify the number of cyanobacterial cells/L. Samples containing over 20,000 cells/mL, equating to the relatively low probability of adverse health effects limit [34], are designated as exceeding the monitoring threshold, and actions are generally taken by the relevant water body owner to prevent public exposure of both humans and animals to the affected water bodies [14,33]. Bloom samples showing cyanobacterial scum formation are automatically designated as exceeding the high probability of adverse health effects threshold, as they may increase cell density and bloom toxicity by up to a factor of 1000 [33,34]. 

Whilst the detection and enumeration of high cell densities of cyanobacterial genus enables water body owners to react to a potential risk, toxin production by blooms is not guaranteed. *Microcystis* blooms may contain either toxin or non-toxin producing strains, and even toxic blooms may vary in the levels of toxin production [34]. Worldwide, typically 40–70% of blooms are reported as being toxic [13,34]. Consequently, whilst any actions taken to close water bodies to the water body users in the event of a dense cyanobacterial bloom may be a sensible precaution, there is the potential for unnecessary closure if the blooms are non-toxic. Such closures are therefore likely to result in socio and economic impacts in at least 40% of cases on average. As such, there are notable benefits for the confirmation of bloom toxicity to enable more focused action to be taken.

Whilst visual examination of cyanobacterial blooms using light microscopy is a useful tool for detection and enumeration of potentially toxin-producing genera, this approach is not sufficient for detection of toxins [35]. As such, toxin testing methods are necessary to investigate the potential toxicity of cyanobacterial populations during bloom incidents, as well as providing an overall understanding of the occurrence of cyanotoxins to facilitate risk assessment and risk management strategies [11]. Toxin detection methods available include bioassays, enzyme and antibody based assays and chemical analytical techniques, such as liquid chromatography [11,24,36,37,38,39], allowing for the quantification of toxicity or toxin levels in environmental samples.

Recently Cefas has developed and validated a rapid liquid chromatography with tandem mass spectrometry (LC-MS/MS) method for the detection of cyanotoxins, including several of the MCs, in water samples as well as concentrated algal material, allowing for direct quantification of cyanotoxin presence from bloom samples [40]. This method has subsequently been accredited by the United Kingdom Standards Authority (UKAS) to ISO 17025 standard. Since the development of this new method and in partnership with the EA, samples found to contain cyanobacteria and therefore suspected of containing cyanotoxins, have been analysed for their toxin content at the Weymouth laboratory of Cefas to provide confirmation of toxin occurrence and aid the overall risk assessment and risk management process. This paper presents the findings from one year of this testing.

## 2. Results

### 2.1. Total Microcystins

#### 2.1.1. Microcystin Occurrence

**Figure 1:**
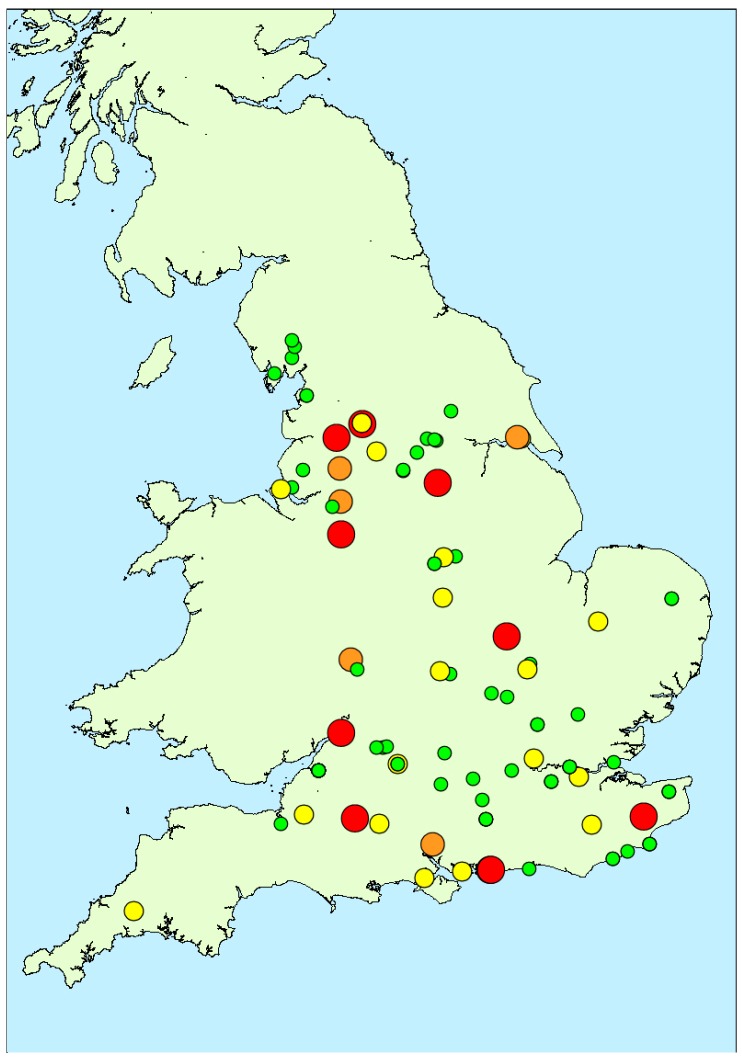
Map showing locations of water samples taken during 2016 study together with total microcystins quantified (red: >100 μg/L; orange: 20–100 μg/L; yellow: 2–20 μg/L; green: <2 μg/L).

Seventy different water bodies were sampled throughout England as part of the EA cyanobacterial response programme, between February and December 2016 inclusive, generating a total of 137 water samples across the country (Figure 1). The sampled water body sizes varied widely, ranging from relatively small lakes and ponds around 10–30 m in length, to large reservoirs and natural lakes over 1 km in length, with total water surface areas ranging from approximately 0.0003 to 5 km^2^ (Table A1). Out of the 137 water samples, 91 were taken from natural lakes, nine from water reservoirs, and the remaining 37 from artificial lakes. Taxonomic identification was conducted in 117 samples in total, with 20 samples not being assessed as the samples were not part of the formal EA response programme and were sent to Cefas directly for toxin testing. Cyanobacterial blooms identified by light microscopy included species of the genera *Microcystis*, *Anabaena*, *Oscillatoria*, *Aphanizomenon*, *Aphanothece*, *Pseudoanabaena*, *Gloeotrichia*, *Gomphospaeria*, *Lyngbia*, *Planktothrix*, *Snowella* and *Merismopedia*. Out of the 137 samples taken, 99 (72% of total) returned cell densities above 20,000 cells/mL including samples with scum, and from this 77 (56% of total samples) were found to contain algal scum floating on the water surface. Samples containing visible scum as thereby exceeding the threshold were consequently not enumerated.

**Figure 2:**
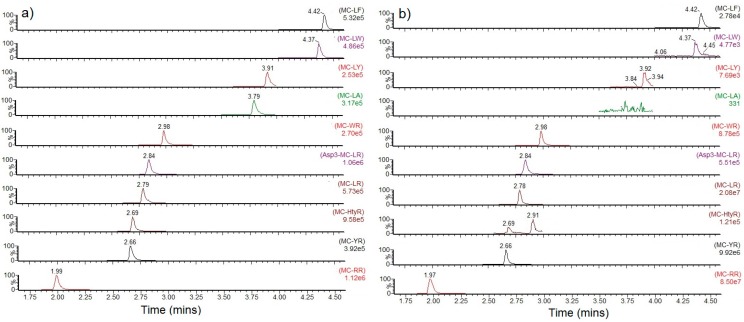
SRM chromatograms for individual microcystin analogues detected in (**a**) high level calibration standard (**b**) sample containing the highest total toxin result (sample 114). MC = Microcystin.

Table A1 also summarises the total microcystin concentrations quantified in each water/bloom sample, above the limit of quantitation (LOQ) of 0.2 μg/L. The total microcystin values include the sum of both intracellular and extracellular concentrations. For the purpose of toxin concentration calculations in terms of pg/cell, samples containing scum were assumed to have a cell density of 500,000 cells/mL (Table A1). From the 137 water samples analysed, 72 (53%) were found to contain total microcystins above 0.2 μg/L. 40 samples (29%) returned total MCs above 2 μg/L which equates to the WHO low probability health alert level [34], whereas a total of 18 (13%) samples contained total MCs above the moderate probability health alert level of 20 μg/L. Three samples were taken from water bodies associated with fish kills (Table A1), although none of these samples were found to contain total MC concentrations above 1 μg/L. Of the 18 samples above the 20 μg/L limit (Table 1), total toxin concentrations were found to vary widely, with one scum-containing sample returning an extraordinarily high total MCs result of 42.7 mg/L (Table 1). In terms of total toxin concentrations per cell, results ranged from approximately 40 pg/cell to 85 ng/cell, although it is recognised this is likely to be an over-estimation given that the assumption of 500,000 cells/mL for all scum containing samples may be under-estimated. Figure 2 illustrates the SRM chromatograms obtained following the analysis of a high-level calibration standard and the bloom sample containing the highest concentrations of toxins. Two other samples reached concentrations above 1 mg/L, with a further seven above 100 μg/L. 17 out of 18 samples (94%) containing total MC above the 20 μg/L were from water bodies with visible scum on the surface, although all 18 samples exceeded the threshold (20,000 cells/mL) and/or contained visible scum (Table 1). Conversely, out of the 99 samples found above threshold of 20,000 cells/mL and/or having visible scum, 39 (39%) were found not to contain microcystins above LOQ, and 42 (42%) samples were below the 20 μg/L medium health alert level. Consequently, out of all samples above the cell density threshold, only 18 (18%) were associated with total microcystin concentrations above the 20 μg/L limit.

#### 2.1.2. Seasonality

**Figure 3:**
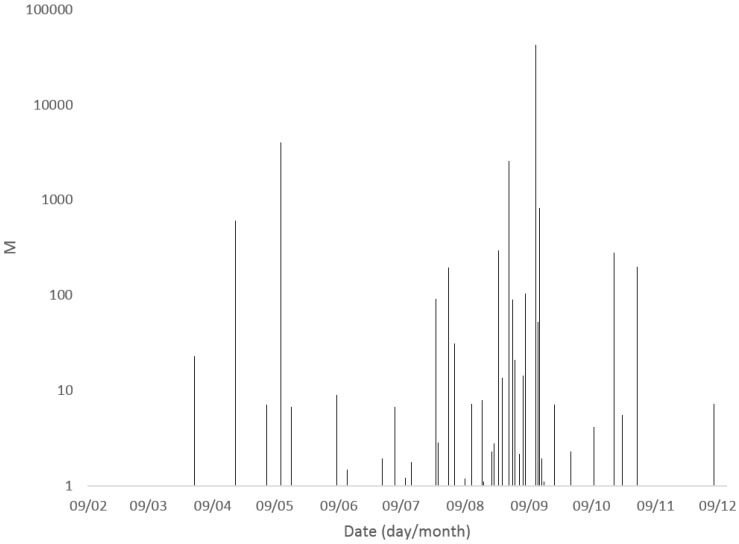
Summary of total microcystin concentrations (extracellular + intracellular) quantified in freshwater samples from England during 2016.

Microcystins were detected and quantified in freshwater bodies throughout England between February and December 2016. Figure 3 illustrates the summed intracellular and extracellular microcystin concentrations determined throughout the year, highlighting a clear dominance in water body bloom toxicity between July and September. Out of the 18 samples containing total MC concentrations above 20 μg/L, 13 were sampled between July and September, including sample 114 which was collected mid-September and contained the highest total toxin concentrations. The earliest recorded sample containing MC above 20 μg/L was taken at the end of March, equating to the beginning of Spring in England, with the latest significant result occurring on the last day of October (Table 1). 

#### 2.1.3. Spatial Variability

**Figure 4:**
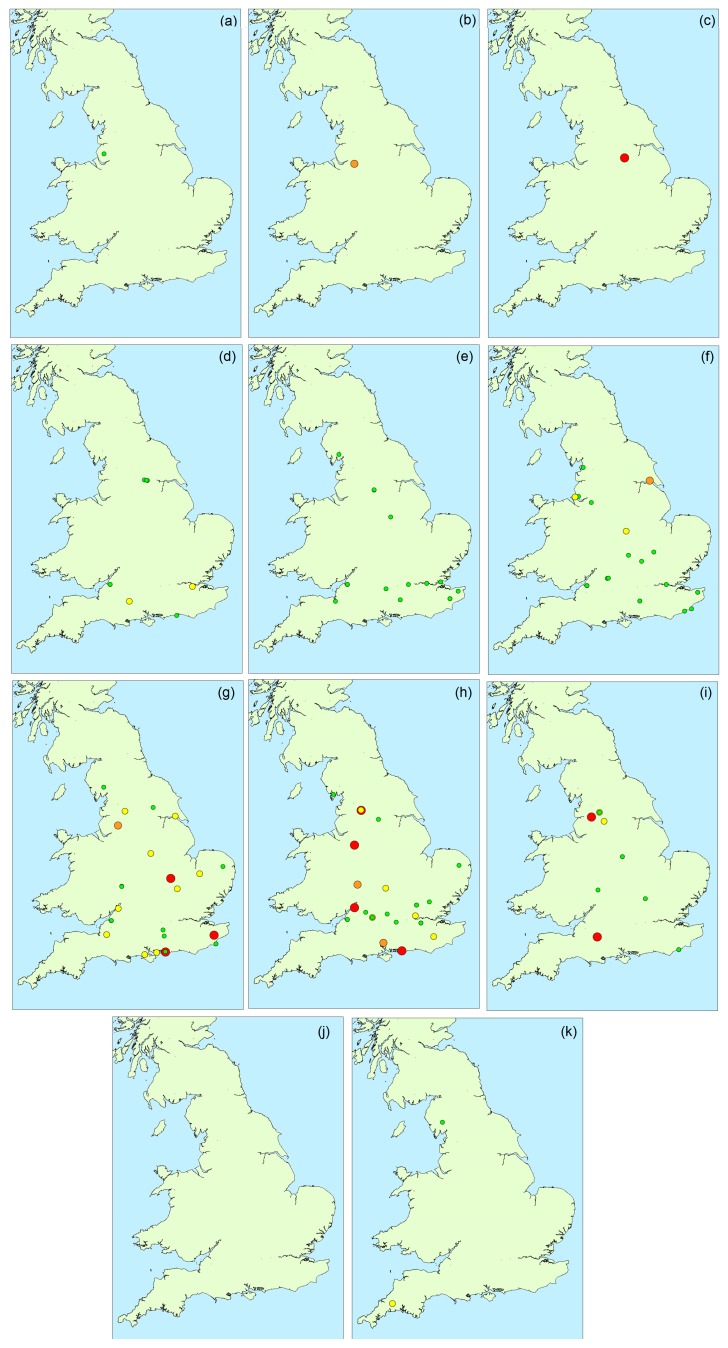
Occurrence and magnitude of total microcystins in water samples throughout England during 2016: (**a**) February; (**b**) March; (**c**) April; (**d**) May; (**e**) June; (**f**) July; (**g**) August; (**h**) September; (**i**) October; (**j**) November; (**k**) December (red: >100 μg/L; orange: 20–100 μg/L; yellow: 2–20 μg/L; green: <2 μg/L).

Figure 4 illustrates the spatial distribution of microcystin positive water samples obtained throughout England during 2016. During the first three months (February to April), only one bloom sample per month was tested, with all three originating from the north of the country. During May and June, more blooms were found in the southern areas, although the majority did not contain microcystins above 2 μg/L. Between July and October, an even spread in bloom occurrence was evident across the country, with high microcystin concentrations determined in samples in both the north and the south, as well as the central and south-eastern regions. Overall, there was no visual indication of any geographically-related patterns of microcystin abundance in the bloom samples received throughout the year.

#### 2.1.4. Taxa

A total of 12 different cyanobacterial genera were identified in the water samples taken and subjected to taxonomic identification, with one additional brackish water sample containing the dinoflagellate *Gymnodinium* (Table A1). The most commonly identified cyanobacterial genus was *Microcystis* in 48 out of the 117 (41%) bloom samples assessed by light microscopy, followed by *Anabaena* in 40 samples (34%), *Aphanizomenon* in 33 samples (28%), *Oscillatoria* in 25 samples (21%) and *Gomphospaeria* in 14 samples (12%). *Planktothrix* and *Gloeotrichia* were both present in 5 samples each (4%) with the remaining genera found in either 1 or 2 samples in total. Overall, 71 samples (61%) contained a single identified genus, with 36 (31%), 7 (6%) and 3 (3%) having two, three and four cyanobacterial genera respectively. Out of the 48 samples containing *Microcystis*, 43 (90%) showed detectable concentrations of microcystins (≥0.2 μg/L), with 13 of these above the WHO 20 μg/L medium health risk threshold (Table 2). Only 17 of the *Microcystis* samples, however, contained *Microcystis* exclusively, with the remaining 31 consisting of mixed genus blooms. Out of these 17 samples, 15 showed detectable concentrations of microcystins, although only three had total MC concentrations above 20 μg/L (Table A1). Other cyanobacteria found as single genus without the presence of other genera included *Gomphospaeria* (3 samples, with total MCs ranging from <LOQ to 199 μg/L), *Anabaena* (17 samples, with three above 1 μg/L, specifically 6.7, 611 and the second highest quantified total concentration of 4019 μg/L), *Aphanizomenon* (17 samples, five >LOQ, with a maximum total MC concentration of 5.5 μg/L), *Oscillatoria* (seven samples, three >LOQ, maximum 7.1 μg/L). *Planktothrix* was present exclusively in just five samples, with total MC concentrations not exceeding LOQ in any. None of the water samples containing *Gloeotrichia*, *Aphanothece* and *Gymnodinium* exclusively were found to contain toxins. As such, there was evidence for significant production (>20 μg/L) of microcystins from single genus blooms containing *Microcystis, Gomphospaeria* and *Anabaena*. From other samples containing more than one cyanobacterial genus, it was impossible to determine the source(s) of the toxins quantified. Sample 114, which recorded the highest toxin concentrations, was associated with a mixed bloom of *Microcystis* and *Gomphospaeria*, whereas the third highest sample, 92, consisted of *Microcystis*, *Anabaena* and *Aphanizomenon*. Overall, out of the 18 samples containing total microcystin concentrations above 20 μg/L, 13 were associated with *Microcystis*, eight with *Aphanizomenon*, seven with *Anabaena*, four with *Gomphospaeria* and two with *Oscillatoria (*Table 1).

#### 2.1.5. Intra and Extra-Cellular Toxins

The vast proportion of toxins in the majority of samples were detected in the intra-cellular fraction, with only low extra-cellular concentrations quantified in a few bloom samples throughout the year. The one notable exception was sample 3, taken in April from NW England, consisting of *Anabaena flos aquae* exclusively. The total microcystin content was 611 μg/L, which consisted of 351 μg/L (57%) as intra-cellular and 260 μg/L as extra-cellular. Other samples found to contain significant proportions of extra-cellular microcystins were those with much lower total toxin concentrations, with a total of four other samples exhibiting more than a 10% extra-cellular toxin proportion. With the sample 3 outlier removed, the mean proportion of intra-cellular toxins to the total microcystin content, in samples containing MCs > 2 μg/L, was 94%. 

#### 2.1.6. Environmental Influences

**Figure A1:**
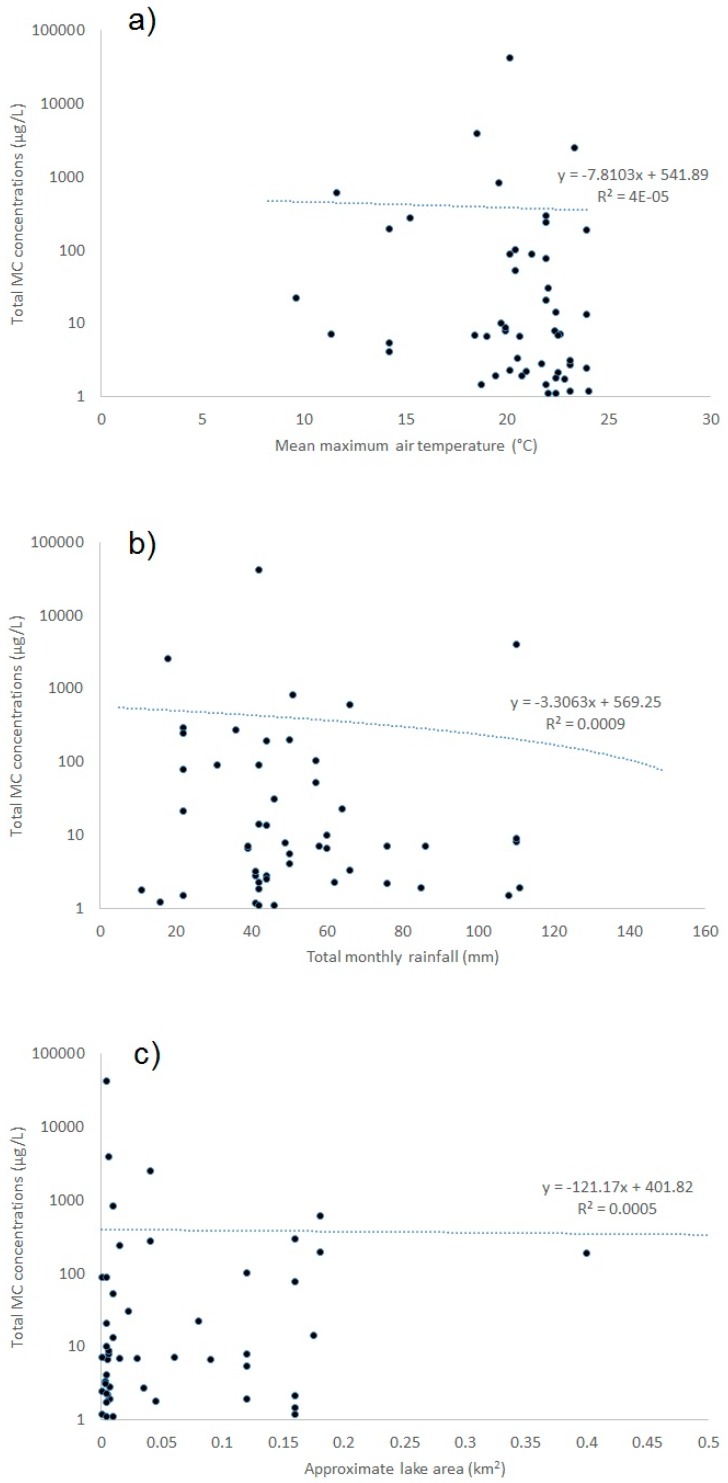
Regressions showing correlation coefficients between environmental factors and total microcystin concentrations (**a**) mean maximum monthly air temperatures; (**b**) total monthly rainfall; (**c**) lake area.

Meteorological conditions were obtained from a UK database and used to assess any potential correlation with the occurrence of microcystins (Figure A1). Mean monthly air temperatures recorded at meteorological stations closest to each sampling site were plotted against total toxin concentrations as quantified by LC-MS/MS. A linear regression was assessed between the two factors and no statistical correlation was found (r^2^ ≤ 0.001). Water samples containing the highest toxin concentrations >100 μg/L, were associated with air temperatures ranging widely between 11 °C and 25 °C. Consequently there was no apparent optimum air temperature associated with maximum toxin production in the samples obtained from this study. Similarly, no statistical correlation was found between air temperature and microcystin toxin concentrations per cell (data not shown). Total rainfall data was also assessed against toxin concentrations, and again no correlation found between the two data sets (r^2^ ≤ 0.001). No statistical correlation was also observed between overall lake size (area) and toxin levels, although visually it was noted that the water samples containing the highest toxin concentrations were associated with the smallest lake areas (r^2^ ≤ 0.001).

### 2.2. Toxin Profiles

#### 2.2.1. Mean Profiles

**Figure 5:**
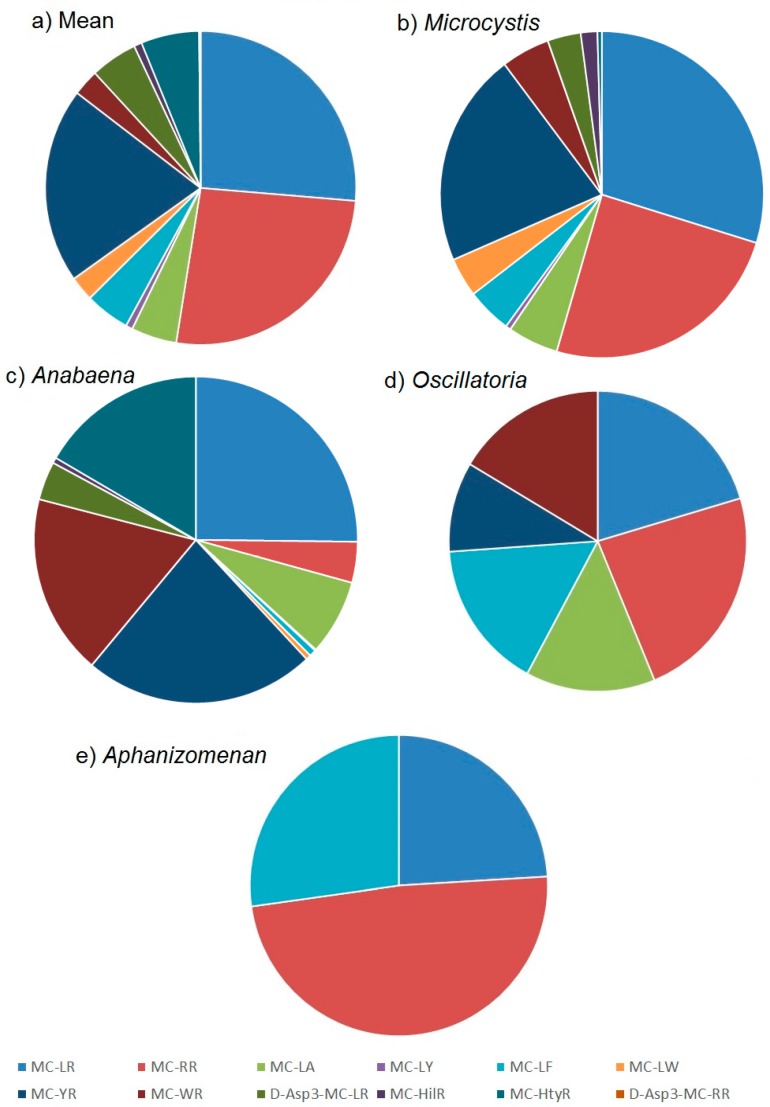
Proportions of microcystin analogues quantified in water samples from England illustrating toxin profiles from (**a**) all samples (**b**) *Microcystis* bloom samples (**c**) *Anabaena (***d**) *Oscillatoria* and (**e**) *Aphanizomenon*.

The quantitation of microcystins in the water samples by LC-MS/MS enabled the determination of toxin profiles in each sample. The mean microcystin profile obtained across all microcystin-positive samples is illustrated in Figure 5a. A wide range of toxin analogues were detected including most notably MC-LR, MC-RR and MC-YR, with each of the three representing approximately 30% of the total toxin content on average. MC-LA, MC-LF, MC-WR, D-Asp3-MC-LR and MC-HtyR were each present at an average of 5% of the total microcystin content, with MC-LY, MC-LW, MC-HilR and D-Asp3-MC-RR present only at trace proportions throughout. This mean profile incorporates the toxin concentrations determined from bloom samples containing a variety of cyanobacterial genera. 

#### 2.2.2. Profile Dependencies

**Figure A2:**
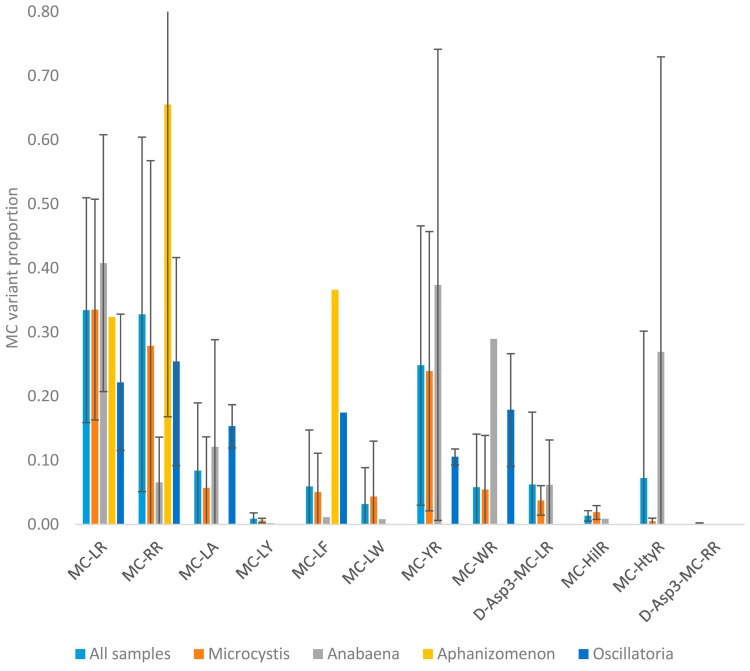
Summary of mean microcystin profiles (±sd) in English cyanobacterial blooms across all samples together with mean profiles (±sd) from single genus blooms.

Whilst 39% of taxonomically assessed water samples were found to contain more than one cyanobacterial genus, the results obtained from the remaining 61% single genus samples enabled a further assessment of toxin profiles to be made in relation to the source cyanobacteria identified. The most commonly occurring *Microcystis* genus showed on average a complex toxin profile, consisting of a wide range of microcystin analogues (Figure 5b). The three dominant microcystins were found to be MC-LR, MC-RR and MC-YR, representing on average 85% of the total toxin content. The remaining toxins consisted of lower proportions of MC-LA, MC-LF, MC-LW and MC-WR, followed by minor concentrations of MC-LY, MC-HilR and MC-HtyR. The variability associated with these mean proportions was found to vary significantly however (Figure A2) with concentrations of the three dominant analogues MC-LR, MC-RR and MC-YR varying from 13% to 63%, 2% to 75% and 4% to 63% of the total toxin concentrations respectively. Single factor ANOVA analysis on profile data confirmed there were statistical differences between the toxin proportions determined in each genus. Toxin profiles determined in bloom samples containing exclusively *Anabaena*, were also found on average to show a dominance of MC-LR and MC-YR, although in contrast to *Microcystis*, lower proportional concentrations of MC-RR and higher amounts of MC-LA, MC-WR and MC-HtyR were quantified (Figure 5c). As with *Microcystis*, the variability around the mean proportions was found to be high, with MC-YR ranging from 2% to 87% of the total toxin content from the samples analysed. In contrast, *Oscillatoria* and to a greater extent *Aphanizomenon*, showed simpler toxin profiles on average, with samples of the latter genus containing a dominance of MC-RR, together with MC-LR and MC-LF (Figure 5e). *Oscillatoria* was found to contain near equal mean proportions of MC-LR, MC-RR, and MC-LF with slightly lower proportions of MC-WR, MC-YR and MC-LA (Figure 5d).

**Figure A3:**
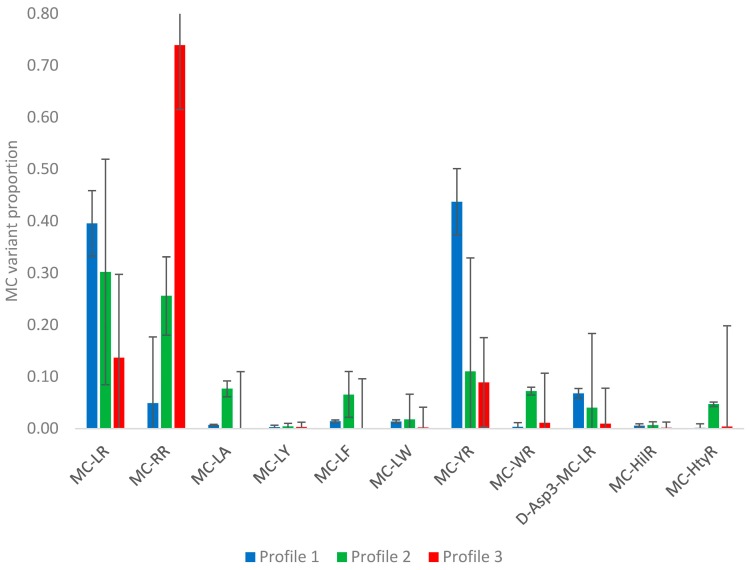
Mean microcystin variant proportions (±sd) determined in each of the three profile types (clusters).

In the absence of any genus-related toxin profile patterns, a *K*-means cluster analysis was conducted using MS Excel algorithms on the variant profile data to assess the presence of any specific profile types from the quantitative data generated. Three specific clusters were identified, relating to three profile types. Figure A3 illustrates the mean microcystin proportions for each of the three profiles determined. Profile 1 was found to consist of high relative proportions of MC-LR and MC-YR, with low relative proportions of all other microcystin variants. On the other hand, profile 2 was found to contain MC-LR and MC-RR as the two dominant toxins together with higher proportions of a larger number of analogues, including MC-LA, MC-LF, MC-YR, MC-WR, D-Asp3-MC-LR and MC-HtyR. Cluster three showed a very different mean profile with a clear dominance of MC-RR and all other toxins present at relatively low proportional concentrations. 

**Figure 6:**
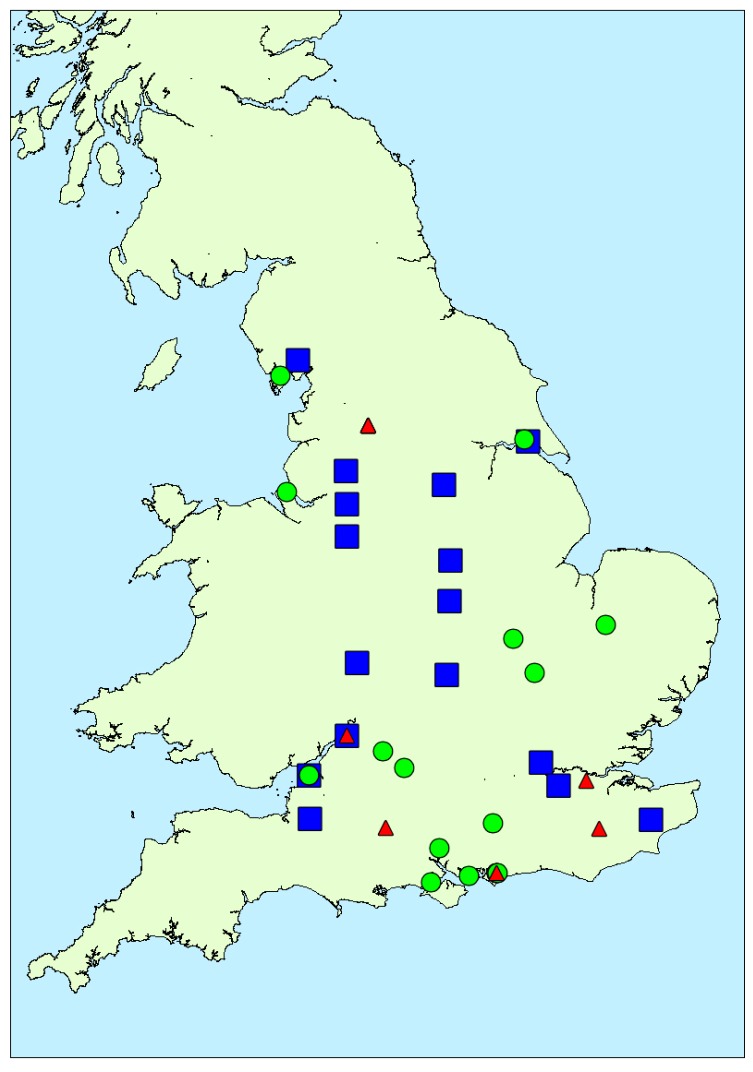
Location of microcystin-positive water bloom samples containing toxin profiles of each profile cluster (blue squares = profile 1; green circles = profile 2; red triangles = profile 3).

**Figure A4:**
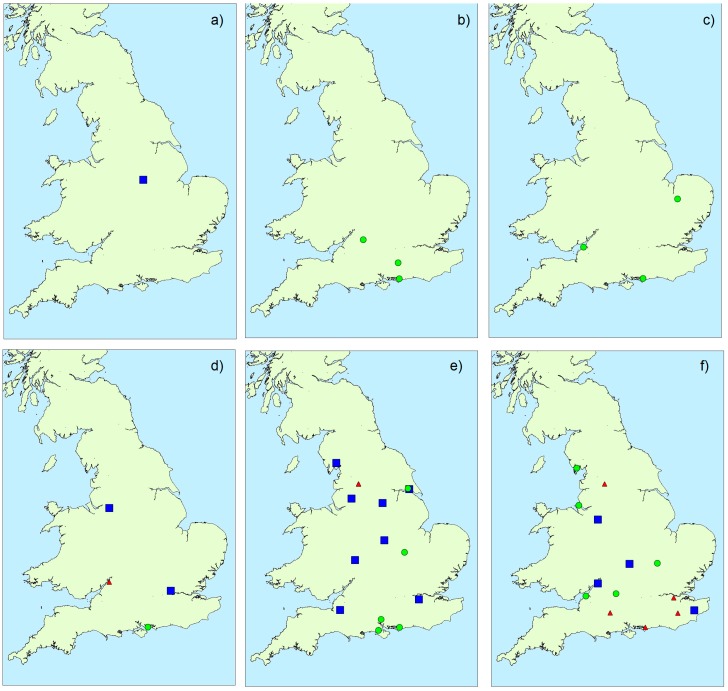
Microcystin profile clusters determined in bloom samples throughout England during: (**a**) April; (**b**) May; (**c**) June; (**d**) July; (**e**) August; (**f**) September (blue squares = profile 1; green circles = profile 2; red triangles = profile 3).

**Figure A5:**
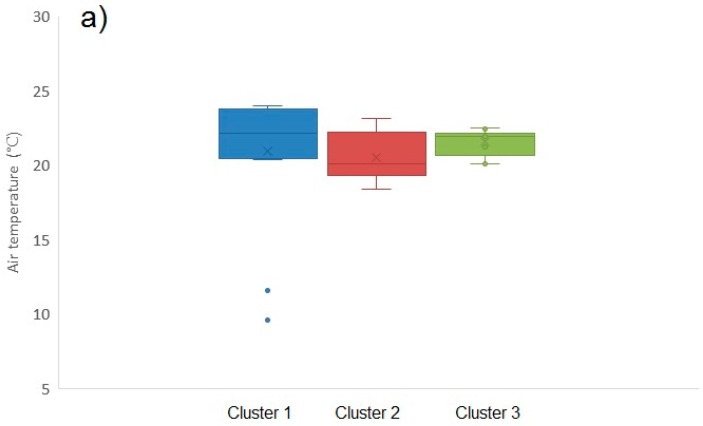

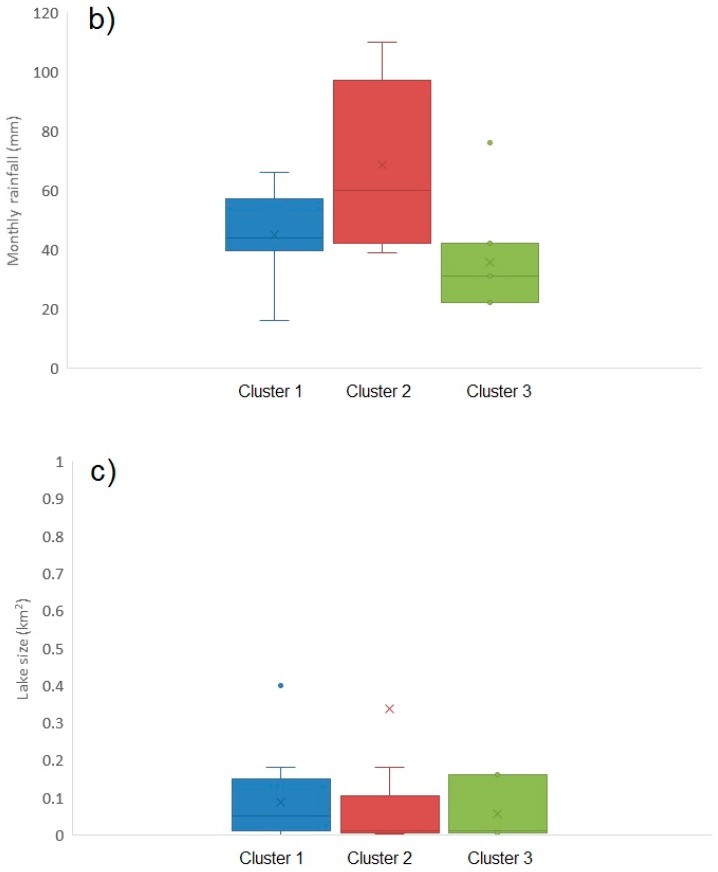
Box and whisker plots illustrating environmental parameters for each of the three toxin profile clusters: (**a**) mean maximum air temperatures (°C); (**b**) total monthly rainfall (**c**) lake size (km^2^).

Figure 6 illustrates the distribution of toxin profile clusters in bloom samples obtained from around the country. Samples associated with profile 1 and profile 2 were found throughout, whereas with one exception profile 3 samples were located in the south. Overall, however, there was little evidence for any geographical impact on toxin profile. Similarly, there was no strong evidence for any temporal association with profile, with the exception of profile 3 occurring later in the year than profile 1 and 2 (Figure A4). Figure A5 illustrates the potential impact of environmental parameters on toxin profiles. Mean air temperatures were found to be lower for samples containing microcystins associated with profile 2 (20.54 ± 1.58 °C), with higher mean temperatures calculated for profile 1 (22.37 ± 1.32 °C) and profile 3 (21.54 ± 0.90 °C) samples. However, with a wide variability of temperatures around the mean, there was no statistical difference found between these profiles in terms of air temperature (single factor ANOVA, F = 0.373; F critical = 3.24; 95% confidence. Similarly, there was no relationship between lake size and profile type (ANOVA, F = 0.57; F critical = 3.24; 95% confidence). However, notably, profile 3 samples were found to be associated with samples taken during months with lower total rainfall amounts (profile 3 mean total rainfall = 35.7 ± 17.7 mm), in comparison to rainfall levels associated with profile 1 (42.0 ± 14.6 mm) and profile 2 (68.8 ± 27.6 mm) samples. A single factor ANOVA assessment confirmed there was a statistical difference between the rainfall levels associated with each of the three profile clusters (F = 8.47; F critical = 3.24; 95% confidence). Conversely, profile 2, containing the greatest number of different MC variants, was associated with water samples taken from areas during periods of the highest rainfall.

## 3. Discussion

For many years, government agencies in the UK have conducted microscopic detection and enumeration of cyanobacterial species in freshwater bodies containing visual algal or bacterial blooms. Water samples found to contain potentially toxin-producing species above a cell density threshold of 20,000 cells/mL or the presence of cyanobacterial scum have triggered action by water body owners to prevent or restrict public access to the water body, thereby reducing the health risk to both humans and animals. In the absence of toxicity data, however, such action may result in the unnecessary closure of lakes and ponds. Conversely, potentially low-density blooms of highly toxic cyanobacterial cells may contain toxins at high enough concentrations to cause health effects, without triggering the cyanobacteria cell density threshold. In addition, there is a scarcity of information describing the spatial and temporal occurrence of toxic blooms in the UK, as well as the concentrations of toxins typically encountered, so the impact on the general public remains unknown. This study aimed to generate data to describe the toxicity of cyanobacterial blooms throughout a year-long period in England, and highlight any patterns of occurrence which could aid the risk assessment and management process.

Overall, LC-MS/MS results showed more than half the water samples contained quantifiable concentrations of microcystins. These findings are not surprising given that these were reactively-sampled, based on visual reports of blooms by water body owners. This proportion therefore concurs with previous reports of 40–70% of global blooms being toxic [1,34] and the UK work conducted in the 1980s reporting 68% of blooms containing toxins [13]. Microcystin concentrations reported here are well above average values reported in the majority of previous studies. Codd et al. found concentrations of microcystins reaching a maximum of 131 μg/L in water from the UK following analysis by HPLC [21], with reports from Germany and Portugal showing maximum toxin concentrations of 36 μg/L and 37 μg/L respectively [41] and water samples from Japan reaching 480, 1300, 15,600 and 19,500 μg/L in a number of different studies [41,42,43]. A recent report describing the application of a LC-MS/MS method for cyanotoxins in natural waters across Europe including France, Italy, Ireland, Germany, described microcystin concentrations <3 μg/L [44]. Some of the water samples from this study contained very high concentrations of microcystins, with a maximum above 40 mg/L. Very high concentrations of microcystins per litre of water have been reported up to 25 mg/L microcystin [34,45], whilst noting that these would be from scums or from very dense accumulations of cyanobacteria. The formation of highly toxic scum with the 1000-fold accumulation in cyanobacterial bloom and toxin concentrations have been well described, notably resulting in an increase in cyanotoxin risk [34,46]. Sivonen, K reported that whilst toxin concentrations vary hugely in water, in bloom conditions milligram amounts of microcystins have been reported. The water samples reported in this study, some of which included large amounts of cyanobacterial scum, were sampled reactively, and in many cases represent a worse-case scenario in terms of total toxin loading. As such, the high concentrations of toxins reported here for some samples must be interpreted with care. During this study, dry weight values of cyanobacterial biomass were not determined, so it is difficult to compare our values directly against those determined elsewhere. In relation to potential health effects, 29% and 13% of water samples were found to exceed the low and medium probability health alert thresholds for recreational water (2.0 and 20 μg/L), respectively. The results therefore demonstrate a significant risk to humans and animals accessing recreational waters, which could potentially increase in the future with predicted changes to climatic change, in particular, temperature and rainfall patterns [2,8,13,20]. 

The data obtained from this study have shown the potential for toxic cyanobacterial blooms to be formed throughout at least nine months of the year, with significant concentrations of microcystins being present as early as March and as late as November. The data show, however, the highest prevalence of bloom formation and toxin production occurred between the narrower window of July to October, representing one third of the year-long study. As such, the period of highest toxicity risk occurred in 2016 during the mid to late summer, extending into early autumn, although the potential for earlier bloom formation should not be discounted. This would fit with the well-established notion that cyanobacterial growth rates and thus bloom occurrence is more widespread during periods when light intensities and water temperatures are higher [1,2,10,11,16,34] and agrees with the findings of [3] who noted the common occurrence of cyanobacterial blooms in late summer during a five year period between 2000 and 20005 in UK water reservoirs. Other previous studies have concluded that the summer months are dominated by green algae, with cyanobacteria succeeding in late summer, autumn and early winter [47]. During August and Sept 2016, the weather was changeable throughout the country, but hot and humid weather was present intermittently, with the UK mean temperature being 2.0 °C above the 1981–2010 long-term average, making it the equal second warmest September on record since 1910 [48]. In addition, there were periods of sustained rain, including heavy thunderstorms bringing localized flooding in some areas of the country, which are likely to have resulted in increased nutrient loading to some water bodies around this time. The higher number of toxic blooms obtained during these two months may therefore relate to both the unseasonably high temperatures as well as the increased levels of rainfall. Whilst microcystin production is generally thought to be fairly constant for any given strain of cyanobacteria [49,50], there are reports of MC variant proportions changing in response to temperature modifications [16]. Consequently, the role of environmental factors in both bloom abundance and toxin production is not fully elucidated [51]. Temperature may well influence not just bloom dynamics, but also influence the preferred production of the toxic fraction of any given cyanobacterial population [1,52,53,54]. Whilst there may be evidence for such a relationship for *Microcystis* sp., there are contradictory data showing an inverse relationship between temperature and toxin production in *Planktothrix* [55]. However, any significant increase in water column temperature and nutrient concentrations could explain the formation of high-density blooms and consequently high toxin concentrations. Further screening work will be required in future years to establish inter-annual variations in bloom formation and toxin production, together with an assessment of environmental inputs before any formalised risk assessment can be performed.

LC-MS/MS data showed the occurrence of high toxicity samples across the entire country, with no indications of any geographical patterns which may link to temperature or any other meteorological parameters. Interestingly, sample 114, which contained extraordinarily high concentrations of microcystins, was taken from a relatively small artificial lake, approximately 100 m × 40 m in size, during September in NW England, an area of the country not traditionally associated with high air temperatures. However, during September, the NW of England experienced maximum and minimum temperatures 2.2 and 2.6 °C higher than the 1961–1990 average, together with higher than expected rainfall and sunshine hours [48]. The relatively small water body would therefore have been subjected to prolonged above-average warming and freshwater/nutrient input, all of which may have potentially heightened the bloom intensity and associated toxin production. However, results indicated there was no statistical correlation between air temperature, rainfall or lake size and total microcystin concentrations, expressed either in terms of total toxins per litre of water or total toxins per bacterial cell. Overall, it is likely that highly specific localized factors such as nutrient concentrations and wind conditions will have influence on the growth and toxicity of cyanobacterial blooms. Further studies would be required in GB to explore these environmental parameters in greater detail.

It is recognised that cell densities enumerated by microscopy and toxin concentrations do not necessarily correlate due to the considerable variation in cell toxin content between cyanobacterial strains [56,57]. As such, the determination of cyanobacterial cell densities is not the best indicator of actual toxin exposure [58]. Over half the samples taken during this study contained some form of visible algal scum, with 72% in total exceeding the cell density threshold. Whilst the inclusion of significant amount of scum is likely to have resulted in the very high microcystin concentrations reported in some water samples, out of these 99 samples only 18% were found to contain total microcystins above the WHO medium health alert guidance limit of 20 μg/L. As a consequence, sole reliance on microscopy identification and enumeration is likely to result in the closure or access restriction to more water bodies than necessary, potentially resulting in impacts on industry, event organisation and public recreation. Conversely, out of the 18 bloom samples containing total microcystins above the 20 μg/L guidance limit, 17 contained a visible algal scum with the other exceeding the cell density threshold. This therefore shows good evidence that the current monitoring regime provides effective protection for humans against recreational water exposure during periods of freshwater algal blooms. Together, these results indicate that whilst a monitoring programme involving microscopy alone appears suitable for protection of human health, the incorporation of toxicity screening using chemical detection methods will provide a more realistic assessment of the risks associated with recreational water bodies, reducing potential future socio-economic impacts relating to water body closures.

The most commonly identified cyanobacterial genera in the 117 samples studied were firstly *Microcystis*, followed by *Anabaena*, *Aphanizomenon*, *Oscillatoria* and *Gomphospaeria*, with *Planktothrix* and *Gloeotrichia* also identified in a low number of samples. These findings therefore agree well with those reported from cyanobacterial blooms in the UK during the 1980s, where *Microcystis, Anabaena Aphanizomenon* and *Oscillatoria* were also the top four most commonly identified taxa [11,13]. Whilst approximately 40% of the samples were found to contain multiple species of cyanobacteria, the remainder of samples containing one genera enabled the first determination of toxin concentrations profiles in a range of natural cyanobacterial species occurring in UK freshwaters. Whilst the presence of significant levels of microcystins is sometimes linked primarily to the presence of *Microcystis* spp., the data reported here show concentrations of toxins above the WHO low and medium probability thresholds in single genera bloom samples containing *Microcystis*, *Gomphospaeria*, *Anabaena*, *Aphanizomenon* and *Oscillatoria*. Consequently, there is the potential for toxin production in a wide range of cyanobacteria found to inhabit UK freshwater ecosystems. Out of the 18 water samples with total MC concentrations above the 20 μg/L threshold, only six contained a single cyanobacterial genus, indicating the greatest risks to be present in bloom samples containing mixed taxa.

To date there have been very few descriptions of microcystin toxin profiles present in natural water samples from the UK. Previously, it has been reported that usually only one or two microcystin variants are dominant in any single strain [45]. Lawton, L.A. et al. reported the presence of a number of different MC variants in two samples from England, taken from central and NE England during 1989 and 1992 respectively, with the former implicated in animal deaths at the time of sampling [59]. *M. aeruginosa* was present in both samples and through a combination of LC-DAD and LC-MS were found to contain MC-LR as the dominant analogue, with lower relative proportions of MC-LY and the more hydrophobic MC-LW and MC-LF in addition to a demethylated analogue of MC-LR. In this study, all of these MC variants were identified with high average proportions of MC-RR and MC-YR being quantified across all samples. In addition, MC-LA, MC-WR, D-Asp3-MC-LR and MC-HtyR were quantified, together with occasional low levels of MC-HilR and D-Asp3-MC-RR. These results therefore increase the number of variants reported to date in natural UK cyanobacterial samples, potentially related to the increase in sensitivity of the current LC-MS/MS instrumentation and the larger number of toxins available as reference material standards. 

LC-MS/MS results showed a wide variety in the relative proportions of microcystin analogues from sample to sample. Notably, toxin profiles differed greatly between cyanobacterial genera, in particular with *Aphanizomenon*, and to a lesser extent *Oscillatoria*, containing simpler profiles in comparison to *Microcystis* and *Anabaena*. Interestingly, both these genera were found to contain the highest mean proportion of the more hydrophobic MC-LF. Further afield in Europe, Pekar et al., 2016 quantified a large number of MC variants from natural lake waters containing *Microcystis* spp. in Sweden, most commonly MC-LR, MC-RR, MC-YR, MC-D-Asp3-LR and D-Asp3-RR, all of which were found in *Microcystis* samples from this study. Similarly, LC-LR, MC-RR and MC-YR were the three most abundant toxins present in two lakes from Greece, although other variants including MC-HilR, MC-WR, MC-LY and various demethyl analogues were also identified [60]. Halinene, K. et al. [61] also reported the LC-MS identification of six MC variants in a number of strains of *Anabaena* sampled from the Baltic Sea. These included MC-LR, as well as MC-HtyR and four demethylated variants. Overall, however, in this study there seemed to be little correlation between cyanobacterial genus and specific toxin profiles, and with profiles reported previously from other geographical areas. Identified profile types were not found to correlate with either water body size or air temperatures. However, the level of rainfall measured at meteorological stations close to each sampling site were found to vary significantly between the three profile types. Specifically, profile 3 samples were found to be associated with areas with significantly lower rainfall, with higher levels of rainfall determined in sites dominated by profile cluster 2. As such, the data suggests that microcystin profiles dominated by MC-RR are associated with water samples occurring during periods of lower rainfall, with high rainfall relating to toxin profiles containing a wide variety of MC analogues, including MC-LR, RR, LA, LF, LW, WR, D-Asp3-LR and HtyR. An overall lack of any clear factors affecting the profile type fits with the published notion that toxin profiles produced by cyanobacteria are highly strain dependent [62], with toxin production regulated at the genetic, cellular and population levels [16]. However, the apparent enhanced production of MC-RR in water bodies associated with lower levels of rainfall in this study, and increased proportions of other analogues during high rainfall, is an observation that needs to be assessed systematically in future studies.

## 4. Materials and Methods

### 4.1. Chemicals and Reagents

Mobile phases were prepared from LC-MS-grade acetonitrile (Fisher Optima, ThermoFisher, Greater London, UK) and water used for LC-MS was obtained in-house. Sample preparation reagents were HPLC grade. Toxin standards used for preparation of calibration solutions (MC-RR, MC-LA, MC-LY, MC-LF, MC-LW, MC-YR, MC-WR, MC-HilR, MC-HtyR, MC-LR, [Asp3] MC-LR and Nodularin) were all obtained from Enzo Life Sciences, Exeter, UK. A certified standard of [Dha7] MC-LR was obtained from the Institute of Biotoxin Metrology, National Research Council Canada (NRCC, Halifax, NS, Canada). Reference standards received as solid films were dissolved in 50% aqueous methanol, to form stock solutions. A mixed stock solution was subsequently prepared by combining aliquots of each stock, followed by further dilutions to create seven-level suite of working calibration standards between 0.33 ng/mL to 327 ng/mL per toxin.

### 4.2. Water Sampling

Water samples were taken by The Environment Agency from algal bloom incidents in fresh waters throughout England as part of their incident response duties, following official sampling protocols [33]. Water bodies sampled included natural lakes, artificial lakes and water reservoirs. Samples were taken from the surface of the water bodies by trained water quality samplers or Environment Officers. Specific sampling sites within each water body were chosen by the samplers to be representative of the worst case situation at that bloom. Where a scum was present this was included in the sample. Clean plastic bottles were used to collect 50–100 mL water to be sent to the testing laboratory. Samples were not preserved. After collection, water samples were sent via same day courier or overnight Royal Mail post for microscope analysis at the Environment Agency Area laboratories. This analysis included taxonomic identification to genus level and cell counts per milliliter (cells/mL). Where samples exceeded the Environment Agency’s incident threshold for cyanobacterial cell counts, they were sent to CEFAS Weymouth for toxin testing. In addition to the routine incident response samples, a few other water samples were included, specifically two commercial samples from a private water body and some repeated sampling from a water body in Bristol to generate data for ongoing toxicity assessment. These additional samples were treated in the same way as the incident response samples. For practical reasons, water samplers were unable to take direct measurements of environmental parameters which may influence cyanobacterial growth and toxicity. To facilitate an assessment of potential environmental impacts, meteorological data was obtained from an on-line database published by the Met Office [63]. This provides monthly mean maximum air temperatures and total recorded rainfall across all meteorological sites in the UK. Grid reference locations for each of the water sampling sites were used to determine the geographically-closest meteorological station present in the database, and meteorological data recorded for each water sample. Approximate water body sizes (length and width) were used to estimate total water body areas (km^2^). Water body depth was not possible to measure as sampling officers are not provided with boats to take samples.

### 4.3. Sample Analysis

#### 4.3.1. Detection of *Cyanobacteria*

All Environment Agency incident response algal samples were analysed at the EA biology laboratories by trained and quality assured analysts using high powered microscopes and Sedgewick Rafter counting cell slides. Samples were mixed before analysis to allow even distribution of cells. If the analysis found any nuisance algae genus present, cell counts were performed and compared with warning thresholds for cyanobacteria. Where a scum was present no count was needed and the sample was considered above threshold. The warning threshold levels used were consistent with guidance levels derived from the WHO guidance [34] and modified by [32]. For cyanobacteria blooms the threshold was 20,000 cells/mL. The routine monitoring and commercial water samples were not subjected to microscopy analysis. Cyanobacterial identification was performed using a variety of identification keys, but predominantly with guidance listed in laboratory protocol documents [33]. Identification was performed against a comprehensive database published by the Natural History Museum, containing 5279 species of freshwater algae of relevance to the British Isles. 

#### 4.3.2. Water Sample Processing

Water samples received for testing were shaken to mix thoroughly before measuring into a graduated centrifuge tube. Typically, 45 mL sample was taken, unless lower volumes were supplied. Samples were first centrifuged at 4500 rpm for 10 min. If there was no evidence for floating cells, then the majority of the supernatant was decanted from the algal pellet, leaving approximately 1 mL behind in the centrifuge tube. A minimum of 1.5 mL of decanted supernatant was filtered through a 0.2 μm syringe filter directly into a LC-MS glass autosampler vial for extra-cellular toxin analysis. The pellet and approximately 1 mL remaining supernatant was subsequently mixed, and quantitatively transferred to a 2 mL Eppendorf tube. Samples were centrifuged at 13,000 rpm for 10 min, after which the supernatant was removed and discarded. Eppendorf tubes containing the remaining pellets were placed into a freezer (<−15 °C) for a minimum of 30 min to help lyse the bacterial cells. After this time, tubes were removed from the freezer and 1.0 mL of 80% MeOH added. Tubes were vortex mixed for 30 s, before leaving for 30 min. After a further 30 s vortex mix, tubes were again centrifuged (13,000 rpm, 10 min) after which the supernatant was filtered through a 0.2 μm syringe filter and transferred to a glass autosampler vial for intra-cellular toxin analysis.

For bloom samples containing visibly floating cells, a filtration method was used in preference to the centrifugation method. Recorded volumes of well-mixed water samples were filtered through a 0.2 μm Teflon filter using a vacuum pump. The filter was removed and placed into a 50 mL centrifuge tube, before placing in a freezer (<−15°C) for a minimum of 30 min. After removing from the freezer, 5.0 mL of 80% MeOH was added and the tube shaken gently to dissolve the intracellular toxins. Filters were left in solvent for 60 min, with occasional mixing, before filtering through a 0.2 μm syringe filter into a glass autosampler vial for intra-cellular toxin analysis.

#### 4.3.3. UHPLC-MS/MS Analysis

Chemical analysis of cyanotoxins was conducted as detailed in [40]. A Waters (Manchester, UK) Acquity UHPLC system coupled to a Waters Xevo TQ tandem quadrupole mass spectrometer (MS/MS) was used with a 1.7 μm, 2.1 × 50 mm Waters Acquity UPLC BEH C18 column in conjunction with a Waters BEH C18 guard cartridge. The column was held at +60 °C, and a 5 μL injection volume utilized, together with mobile phase flow rate of 0.6 mL/min. Mobile phase A1 consisted of water +0.025% formic acid, mobile phase B1 comprised acetonitrile (MeCN) +0.025% formic acid. The UHPLC gradient started at 98% A1, dropping to 75% A1 at 0.5 min holding until 1.5 min, dropping further to 60% A1 at 3.0 min, decreasing further to 50% A1 at 4 min, before a sharp drop to 5% A1 at 4.1 min, holding until 4.5 min before increasing back to 98% A1 for column equilibration at 5 min for a further 0.5 min. Each instrumental sequence started with a series of instrumental blanks, followed by toxin calibration standards and a microcystin chromatographic retention time marker solution. The MS/MS source parameters and Selected Reaction Monitoring (SRM) transitions were exactly as specified in [40]. Quantitation of microcystins was performed against external calibration standards with results calculated in terms of μg/L of water.

## Figures and Tables

**Table 1:** Summary of English bloom samples, identified taxa, and total microcystin concentrations (extra and intracellular combined) in samples containing total concentrations above 20 μg/L.

Sample ID	Date Collected	Taxa	Total MCs (μg/L)	Total MCs (pg/Cell) *
2	31/03/16	Ap, Os	22.7	45
3	20/04/16	An	611	1221
P-1	12/05/16	An	4019	na
50	26/07/16	M	91	182
59	01/08/16	M	194	387
63	04/08/16	Ap, M	31	62
99	25/08/16	Ap, M	79	157
101	25/08/16	M	297	594
92	30/08/16	An, Ap, M	2561	5122
94	01/09/16	Go, M	89.9	180
98	02/09/16	An, M	21	42
103	07/09/16	Ap, M	103	206
111	12/09/16	An, M	244	488
114	12/09/16	Go, M	42724	85448
110	13/09/16	An, Ap, M	53	105
119	14/09/16	An, Ap, M	830	1661
134	20/10/16	Ap, Go, Os	279	558
139	31/10/16	Go	199	398

na = not analysed (taxonomy not performed). M = *Microcystis*, An = *Anabaena*, Ap = *Aphanizomenon*, Os = *Oscillatoria*, Go = *Gomphosphaeria*. Assuming scum-containing samples have cell density of 500,000 cells/mL. * = assuming scum-containing samples have cell count of 500,000 cells/mL.

**Table 2:** Summary of the total number of water samples containing each cyanobacterial genus, together with those containing total microcystin concentrations above LOQ, 2, 20 and 100 μg/L.

Total Samples	M	An	Ap	Os	Go	Pl	Gl	At	Ly	Ps	Sn	Me	Gy
	48	40	33	25	14	5	5	2	1	2	1	1	1
No. MC detected	43	22	20	13	13	1	2	1	1	1	0	0	0
No. MC > 2 μg/L	27	9	12	8	8	0	0	1	0	0	0	0	0
No. MC > 20 μg/L	13	7	8	2	4	0	0	0	0	0	0	0	0
No. MC > 100 μg/L	7	4	4	0	3	0	0	0	0	0	0	0	0

M = Microcystis, An = Anabaena, Ap = Aphanizomenon, Os = Oscillatoria, Pl = Planktothrix, Go = Gomphosphaeria, Gl = Gloeotrichia, Gy = Gymnodinium, Ly = Lyngbia, At = Aphanothece, Ps = Pseudoanabaena, Sn = Snowella, Me = Merismopedia.

## Appendix A

**Table A1:** Summary of freshwater sample collection dates, locations, water body types, identified taxa, scum presence, enumerated cells in comparison to 20,000 cells/mL threshold and total microcystin concentrations (extra and intracellular combined) during 2016 throughout England.

Sample	Date Collected	Grid Reference	Size (km^2^)	Type	Taxa	Scum	Threshold Exceeded	Total MCs (μg/L)	Total MCs (pg/Cell ^‡^)
1	09/02/16	SD4648304966	0.0024	NL	M	N	N	0.0	304
2	31/03/16	SJ7500781064	0.08	NL	Ap Os	Y	y	22.7	45
3	20/04/16	SK4764995980	0.18	R	An	Y	y	610.5	1221
4	05/05/16	SU0415037183	0.03	NL	Os	Y	y	7.1	14
5	09/05/16	ST5805477480	0.009	NL	-	N	n	0.0	na
6	09/05/16	TQ1730003400	0.009	NL	Os	N	n	0.0	0.0
P-1	12/05/16	ST4637977160	0.006	AL	An	N	Y	4019.4	na
P-2	12/05/16	ST4637977160	0.006	AL	An	Y	Y	0.8	na
P-3	12/05/16	ST4637977160	0.006	AL	M	N	N	0.8	na
P-4	12/05/16	ST4637977160	0.006	AL	M	Y	Y	0.0	na
7	17/05/16	ST5805477480	0.009	NL	-	N	n	0.0	na
9	17/05/16	TQ5453672982	0.005	NL	Os	N	n	6.7	67,182
8	18/05/16	TQ5453672982	0.005	NL	Os	N	n	0.0	0.0
10	23/05/16	ST5805477480	0.009	NL	-	N	n	0.7	na
11	23/05/16	SE3999828451	0.06	NL	Pl	n	y	0.0	0.0
12	23/05/16	SE4710227289	0.09	NL	Os	n	y	0.0	0.0
13	23/05/16	SE4535127829	0.18	NL	Os	n	y	0.0	0.0
14	03/06/16	ST5805477480	0.009	NL	-	N	n	0.1	na
15	08/06/16	ST5805477480	0.009	NL	-	N	n	0.0	na
16	08/06/16	SK6175739286	0.4	AL	Os An	N	y	0.04	0.3
P-5	08/06/16	ST4637977160	0.006	AL	M	N	N	8.0	668
P-6	08/06/16	ST4637977160	0.006	AL	M	Y	Y	9.0	751
17	10/06/16	TR0318143471	0.04	NL	An	N	y	0.0	0.0
18	12/06/16	SE2221504615	0.09	R	Pl	Y	y	0.2	0.4
19	12/06/16	SE2204504797	0.09	R	Pl	Y	y	0.1	0.3
21	13/06/16	SD3754089840	5	NL	An Os	Y	y	1.5	3.0
20	14/06/16	ST5805477480	0.009	NL	-	N	n	0.8	na
22	17/06/16	TR0318143471	0.04	NL	An	N	y	0.0	1.7
23	21/06/16	TQ4769380228	0.03	AL	Ap Go	Y	y	1.0	2.0
26 *	23/06/16	TQ8153583675	0.0012	AL	An	Y	y	0.0	0.0
29	24/06/16	SU5071867008	0.0225	NL	Ap	Y	y	0.0	0.0
24	26/06/16	ST5805477480	0.009	NL	-	N	n	0.0	na
30	26/06/16	SU8478040480	0.16	NL	M Gl	Y	y	0.4	0.8
25	28/06/16	ST5805477480	0.009	NL	-	N	n	0.0	na
27	28/06/16	TQ4769380228	0.03	AL	Ap	Y	y	0.5	1.0
28	28/06/16	TQ0390677601	0.01	NL	An	Y	y	0.6	1.2
31	30/06/16	ST2975437518	0.0075	AL	Os	Y	y	1.9	3.9
32	30/06/16	TR2268161365	0.004	AL	Os An	Y	y	0.1	0.2
33	04/07/16	TQ4769380228	0.03	AL	Ap	Y	y	0.0	0.0
34	04/07/16	ST5805477480	0.009	NL	-	N	n	0.0	na
35	06/07/16	SK5215608209	0.09	NL	An	Y	y	6.7	13
43 ^†^	06/07/16	TQ9163916682	0.0003	NL	Gy	N	n	0.0	0.0
36	07/07/16	TR2268161365	0.004	AL	Os An	N	n	0.1	92
37 *	08/07/16	TL1775658154	0.03	NL	An	Y	y	0.0	0.3
38	11/07/16	SU8478040480	0.16	NL	M Gl	Y	y	1.2	2.4
41	12/07/16	SJ3791791961	0.0075	NL	An	N	n	0.9	18133
39	13/07/16	ST5805477480	0.009	NL	-	N	n	0.0	na
40	14/07/16	TR2268161365	0.004	AL	An Os	Y	y	1.8	13
42	14/07/16	TQ4769380228	0.03	AL	Ap	Y	y	0.0	0.0
44	18/07/16	ST5805477480	0.009	NL	-	N	n	0.0	na
45	18/07/16	SD4881461153	0.0003	NL	An	Y	y	0.0	0.0
46	21/07/16	ST5805477480	0.009	NL	-	N	n	0.0	na
47	24/07/16	ST5805477480	0.009	NL	-	N	n	0.7	na
48	25/07/16	SJ6844577250	0.125	NL	M An	n	n	0.1	10
52	25/07/16	SU0686094628	0.24	NL	Gl	y	y	0.0	0.0
53	25/07/16	SP5732550449	0.006	NL	Ap	y	y	0.0	0.0
50	26/07/16	TA0848030328	0.0005	AL	M	y	y	90.9	182
54	26/07/16	SD4881461153	0.0003	NL	An	y	y	0.0	0.0
55 *	26/07/16	TR0808521709	0.0008	AL	M An	n	y	0.5	0.1
49	27/07/16	SP8896035736	0.09	NL	Ap	y	y	0.1	0.3
51	27/07/16	SU8478040480	0.16	NL	Gl	y	y	0.0	0.0
56	27/07/16	SJ2926290282	0.0075	AL	M	n	n	2.8	234
57	28/07/16	SU0927095489	0.0024	NL	An	n	y	0.0	0.0
58	01/08/16	ST5805477480	0.009	NL	-	N	n	0.0	na
59	01/08/16	SP9990079600	0.4	NL	M	Y	y	193.6	387
60	01/08/16	SE5809049247	0.0225	NL	An	Y	y	0.0	0.0
61	02/08/16	TR0808521709	0.0008	AL	M	N	y	0.6	0.2
62	04/08/16	TA1094628599	0.0036	AL	An Os	N	y	3.3	62
63	04/08/16	SD7415406707	0.0225	AL	Ap M	Y	y	30.8	62
64	05/08/16	SD4017798465	5		An	Y	y	0.2	0.3
65	09/08/16	TR0808521709	0.0008	AL	M	N	n	1.2	167
67	09/08/16	TR0318143471	0.04	NL	Ap	Y	y	0.1	0.2
66	11/08/16	ST5805477480	0.009	NL	-	N	n	0.0	na
68	12/08/16	SK5282738907	0.06	NL	An M	Y	y	7.2	14
81	15/08/16	SO8307960032	0.00125	AL	An Os	N	y	0.0	0.0
82	15/08/16	SO8315359973	0.025	AL	An Os	N	y	0.0	0.0
80	17/08/16	SO7499006989	0.12	NL	Ap M An At	Y	y	7.9	15.8
78	18/08/16	ST5805477480	0.009	NL	-	N	n	0.0	na
85	18/08/16	SD9089540319	0.004	AL	M Go	N	y	1.1	2.2
79	19/08/16	TG2522007900	0.2	NL	Ap M An	N	n	0.1	1046
83	22/08/16	TR0318143471	0.04	NL	Ap	Y	y	0.8	1.6
86	22/08/16	ST4679744377	0.005	AL	An M Os	Y	y	2.3	4.5
84	23/08/16	ST5805477480	0.009	NL	-	N	n	0.0	na
88	23/08/16	SZ3777596540	0.035	NL	M	Y	y	2.8	5.6
87	25/08/16	SD9089540319	0.004	AL	M Go	Y	y	10.0	20
89	25/08/16	SU8478040480	0.16	NL	Gl	Y	y	0.0	0.0
90	25/08/16	SU8201355304	0.09	NL	Ap	Y	y	0.0	0.0
99	25/08/16	SU8728403186	0.16	NL	M Ap	Y	y	78.7	157
101	25/08/16	SU8728403186	0.16	NL	M	Y	y	296.9	594
102	25/08/16	SU8728403186	0.16	NL	M Ap	N	y	1.5	3.0
100	26/08/16	SU8728403186	0.16	NL	Ap	Y	y	0.1	0.2
91	27/08/16	TL1572653882	0.01	NL	M	Y	y	13.6	27
92	30/08/16	TR0318143471	0.04	NL	Ap M An	Y	y	2561.0	5122
93	30/08/16	TL6943690325	0.0009	AL	M	Y	y	2.5	5.0
97	30/08/16	SU6663501299	0.0036	NL	Ap Ps	N	y	3.2	21
94	01/09/16	SD9089540319	0.004	AL	M Go	Y	y	89.9	180
95	02/09/16	ST5805477480	0.009	NL	-	N	n	0.0	na
98	02/09/16	SU4410721883	0.004	NL	M An	Y	y	21.1	42
96	04/09/16	TQ2097086918	0.16	R	Ap	Y	y	2.2	4.4
107	06/09/16	SU1762682163	0.175	NL	M	Y	y	14.2	28
108	06/09/16	SU1762682163	0.175	NL	Ap	N	n	0.0	0.0
103	07/09/16	SO7499006989	0.12	NL	M Ap	Y	y	103.2	206
104	07/09/16	TQ3403669313	0.0096	NL	Ap	Y	y	0.0	0.0
106	07/09/16	SU5296391042	0.0024	NL	At	N	y	0.0	0.1
105	09/09/16	SE3248717960	0.3	NL	M Ap	Y	y	0.3	0.6
111	12/09/16	SU8779803166	0.015	NL	M An	Y	y	244.0	488
114	12/09/16	SD9089540319	0.004	AL	M Go	Y	y	42,724.1	85,448
110	13/09/16	SO8267761829	0.01	AL	Ap An M	Y	y	52.7	105
112	13/09/16	TL538202	0.0075	NL	An	Y	y	0.2	0.5
115	13/09/16	SU7483471048	0.015	NL	An Ps Os	N	y	0.0	0.0
118	13/09/16	SU1762682163	0.175	NL	Ap	Y	y	0.2	0.3
109	14/09/16	ST5805477480	0.009	NL	-	N	n	0.2	na
119	14/09/16	SJ7493757095	0.01	NL	An M	Y	y	830.4	1661
122	14/09/16	TL 23456 12000	0.01	NL	M	N	n	0.3	2706
116	14/09/16	SU0158594707	0.045	AL	M An	Y	y	1.8	3.7
113	15/09/16	SD2430078100	0.18	R	Go M An	Y	y	1.0	2.0
117	15/09/16	SP4976352470	0.12	R	Ap M An Os	Y	y	1.9	3.9
120	16/09/16	TQ3403669313	0.0096	NL	Ap	Y	y	1.1	2.2
123	19/09/16	TL 23456 12000	0.01	NL	M	N	n	1.0	9926
121	20/09/16	TG2522007900	0.2	NL	Sn Os M Me	N	n	0.0	0.0
125	21/09/16	TQ6421836460	0.015	NL	Os Go	Y	y	7.1	14.2
124	22/09/16	ST5805477480	0.009	NL	-	N	n	0.0	238
126	22/09/16	TQ3403669313	0.0096	NL	Ap	Y	y	0.0	0.1
128	29/09/16	SD9089540319	0.004	AL	Go M	Y	y	2.3	4.6
132	07/10/16	TL0072233302	0.0015	NL	An	Y	y	0.3	5.7
133	10/10/16	SD9089540319	0.004	AL	Go M	Y	y	4.1	8.3
129	11/10/16	SO8745754176	0.0008	AL	Os M	Y	y	0.0	0.0
130	11/10/16	SK4542133791	0.025	NL	Pl	N	y	0.0	0.0
131	11/10/16	SK4542133791	0.025	NL	Pl	N	y	0.0	0.0
135	13/10/16	TQ8074010542	0.002	NL	Go Os Ly	N	y	0.4	1.2
136	13/10/16	TQ8032210477	0.004	R	Go	N	y	0.7	0.8
134	20/10/16	ST8529642290	0.04	NL	Go Ap Os	Y	y	278.8	558
137	24/10/16	SE0209118762	0.12	R	Ap	Y	y	5.5	11.0
138	26/10/16	SD9089540319	0.004	AL	M Ap	N	n	0.5	309
139	31/10/16	SD7146329903	0.18	R	Go	Y	y	198.8	398
140	07/12/16	SX1799070900	0.001	NL	M Os	Y	y	7.2	14
141	13/12/16	NY3750003300	3.2	NL	Go	Y	y	0.0	0.0

* Samples taken from water bodies associated with fish kills, nd = not detected, MCs = microcystins; ^†^ Brackish water, ^‡^ Assuming scum containing samples have 500,000 cells/mL; NL = Natural lake, AL = artificial lake, R = reservoir, na = no analysis. Cyanobacterial genus abbreviations as follows: M = *Microcystis*, An = *Anabaena*, Ap = *Aphanizomenon*, Os = *Oscillatoria*, Pl = *Planktothrix*, Go = *Gomphosphaeria*, Gl = *Gloeotrichia*, Gy = *Gymnodinium*, Ly = *Lyngbia*, At = *Aphanothece*, Ps = *Pseudoanabaena*, Sn = *Snowella*, Me = *Merismopedia*.
